# Comparison of severity of illness scoring systems for patients with nosocomial bloodstream infection due to *Pseudomonas aeruginosa*

**DOI:** 10.1186/1471-2334-6-132

**Published:** 2006-08-17

**Authors:** Alexandre R Marra, Gonzalo ML Bearman, Richard P Wenzel, Michael B Edmond

**Affiliations:** 1 Division of Infectious Diseases, Universidade Federal de São Paulo, Brasil (UNIFESP-EPM)/Hospital São Paulo (HSP), Brazil; 2 Department of Internal Medicine, Medical College of Virginia Campus, Virginia Commonwealth University, Richmond, Virginia, USA

## Abstract

**Background:**

Several acute illness severity scores have been proposed for evaluating patients on admission to intensive care units but these have not been compared for patients with nosocomial bloodstream infection (nBSI). We compared three severity of illness scoring systems for predicting mortality in patients with nBSI due to *Pseudomonas aeruginosa*.

**Methods:**

We performed a historical cohort study on 63 adults in intensive care units with *P. aeruginosa *monomicrobial nBSI.

**Results:**

The Acute Physiology, Age, Chronic Health Evaluation II (APACHE II), Sequential Organ Failure Assessment (SOFA), and Simplified Acute Physiologic Score (SAPS II), were calculated daily from 2 days prior through 2 days after the first positive blood culture. Calculation of the area under the receiver operating characteristic (ROC) curve confirmed that APACHE II and SAPS II at day -1 and SOFA at day +1 were better predictors of outcome than days -2, 0 and day 2 of BSI. By stepwise logistic regression analysis of these three scoring systems, SAPS II (OR: 13.03, CI95% 2.51–70.49) and APACHE II (OR: 12.51, CI95% 3.12–50.09) on day -1 were the best predictors for mortality.

**Conclusion:**

SAPS II and APACHE II are more accurate than the SOFA score for predicting mortality in this group of patients at day -1 of BSI.

## Background

*Pseudomonas aeruginosa *has the highest crude mortality (39%) among bacteria causing nosocomial BSI [1]. Some small studies have evaluated the effect of hospital pathogens in relation to clinical outcome [2,3]. However, there is no consensus regarding the best scoring system for evaluating prognosis in BSI.

Since the development of the APACHE (Acute Physiological and Chronic Health Evaluation) II score [4], many studies of infectious diseases have used this scoring system to characterize the patient's severity of illness [2,3,5]. Several acute illness severity scores have been proposed for evaluating patients on admission to intensive care units, but these have not been compared for patients with nosocomial bloodstream infection (nBSI).

The prognostic value of the APACHE II score [4] at admission to the intensive care unit has been demonstrated. However, it has been shown that progression to organ dysfunction in patients with *P. aeruginosa *infection is an ominous sign [5]. The Simplified Acute Physiology Score (SAPS II) was also developed to be used on admission to the intensive care unit [6]. Another scoring system, the Sequential Organ Failure Assessment (SOFA) score, assesses the incidence and severity of organ dysfunction in critically ill patients [7]. Most studies of serious infectious diseases use one of these scoring systems to assess illness severity [8,9]. However, studies evaluating the outcome of patients with *P. aeruginosa *BSI have relied on a single analysis of APACHE II to calculate mortality risk [10,11].

The purpose of our study was to compare three severity of illness scoring systems for predicting mortality in ICU patients with nBSI due to *Pseudomonas aeruginosa*.

## Methods

### Setting

The Virginia Commonwealth University Medical Center (VCUMC) is an 820-bed tertiary care facility in Richmond, Virginia. The hospital houses 9 intensive care units (ICUs), including pediatric ICUs and a burn unit. Approximately 30,000 patients are admitted annually.

### Study design

Using the Surveillance and Control of Pathogens of Epidemiological Importance (SCOPE) database of bloodstream infections occurring at 49 U.S. hospitals [12], we identified all patients with a nosocomial BSI due to *P. aeruginosa *at VCUMC from 1 January 1996 through 31 December 2003. Patients were considered to have had BSI due to *P. aeruginosa *if ≥ 1 blood culture was positive for this organism. Only monomicrobial BSI in ICU patients were included. Second episodes were excluded. Clinical data were concurrently collected by infection control practitioners using a standardized case report form. The data that were collected routinely included age, gender, duration of hospitalization in the ICU prior to onset of BSI, predisposing clinical conditions, and bloodstream pathogen. Sources of secondary BSI were identified by cultures obtained from distant sites that yielded the same pathogen. Underlying disease was measured by the Charlson weighted comorbidity index, dichotomized into scores of <3 and ≥3 points. Adequate empiric antimicrobial treatment was defined as therapy administered within 24 hours after blood culture samples were obtained that included the administration of any antimicrobial agent to which the *P. aeruginosa *was susceptible [13]. The single exception to the definition was when a susceptible aminoglycoside was used either alone or in conjunction with another antimicrobial to which the organism was resistant. The APACHE II, SOFA and SAPS II scores (Table 1) were calculated retrospectively from 2 days prior through 2 days after the first positive blood culture. The most abnormal value for each parameter in each 24-hour period was recorded. For a single missing value (which occurred sometimes for bilirubin concentrations), a replacement was calculated using the mean value of the result preceding, and the result after, the missing one. When more than one consecutive result was missing, it was considered a missing value in the analysis.

**Table 1:** Comparison of variables collected in APACHE II, SAPS II and SOFA scores.

Variables	**APACHE II**	**SAPS II**	**SOFA**
**Physiological values**			
**Age, years**	X	x	
**Temperature**	X	x	
**Mean arterial pressure**	X		
**Systolic BP**		x	
**Vasopressor**			x
**Heart rate**	X	x	
**Respiratory rate**	X		
**Glasgow Coma Score (GCS)**	X	x	x
**Urinary output (L/day)**		x	x**
**Laboratory parameters**			
**Oxygenation A-aDO_2 _or PaO_2_**	X		
**PaO_2_**		x*	x
**Arterial pH**	X		
**Serum sodium**	X	x	
**Serum potassium**	X	x	
**Serum creatinine**	X		x**
**Serum urea level**		x	
**Hematocrit**	X		
**White blood count**	X	x	
**Serum HCO_3_^- ^(venous)**	only if no ABGs	x	
**Bilirubin level**		x	x
**Platelets**			x
**Chronic diseases/comorbidities**			
**Chronic diseases**		x	
**Chronic health points (APACHE II)/type of admission(SAPS II)**	X	x	

APACHE II score ranges 0–71; SAPS II score ranges 0–146; SOFA score ranges 0–24.

x* If ventilated or continuous pulmonary arterial pressure.

x** Creatinine or urine output.

ABG – Arterial Blood Gas

### Microbiological methods

Blood cultures were processed at the VCUMC clinical laboratory. Blood cultures (each consisting of aerobic and anaerobic bottles) were processed using the BACTEC^® ^9240 blood culture system (Becton Dickinson, Sparks MD). Identification of *P. aeruginosa *and antibiotic susceptibility testing were performed by the Vitek method (bioMérieux).

### Statistical analysis

For continuous variables, mean values were compared using two sample t-tests for independent samples. Differences in proportions were compared using a chi-square test (χ^2^) or Fisher's Exact Test when appropriate. Mean values are reported ± 1 SD. A computation of the area under a receiver operating characteristic (ROC) curve for APACHE II, SAPS II and SOFA was performed at days -2, -1, 0, 1 and 2 of BSI. The sensitivity, specificity, overall correctness of prediction, and positive and negative predictive values for APACHE II, SAPS II and SOFA scores were determined. The cut-off points for predicting in-hospital mortality were identified as the score giving the best Youden index (sensitivity + specificity - 1) for each scoring system on the day with the best discriminating power. The Youden index evaluates the diagnostic efficacy of a test. If the index is equal to or less than 0, the diagnostic efficacy of the test is poor. On the other hand, the closer it is to 1, the higher is its diagnostic value. Odds ratios were calculated for variables associated with crude mortality. Ninety five percent confidence intervals were calculated for all odd ratios. Alpha was set at 0.05. To identify which of the 3 scales best predicted BSI outcome, three different multivariable logistic regression models were built. All statistical analyses were done using the Statistical Package for the Social Sciences software (SPSS, Chicago, IL, USA).

## Results

### Study population and patient characteristics

During the study period, a total of 160 nosocomial *P. aeruginosa *BSIs were identified. Of these, 19 cases (11.9%) were identified in pediatric patients (<18 years of age), 49 were polymicrobial BSI, and 15 cases were monomicrobial BSIs with incomplete medical records. Fourteen patients had infections that were acquired outside the intensive care unit (ICU) setting. The remaining 63 monomicrobial episodes of BSI caused by *P. aeruginosa *in ICU patients were included in the analysis.

Patients included in this study had a mean age of 56 ± 16.4 years (range 19 – 89 years). Sixty percent of patients were male. The most frequent diagnoses responsible for hospitalization were trauma (including burn) (27.0%), gastrointestinal (23.8%), and solid and hematologic malignancies (19.1%). Among the potential factors predisposing to BSI, central intravascular devices were the most frequent (88.9%). In 46 patients (73.0%) ventilatory support was needed prior to the onset of BSI, as was hemodialysis in 12 (19.0%). The most frequent sources of BSI were respiratory (31.7%) and central venous catheter (20.6%). More than one-half of BSIs (55.6%) occurred after 21 days of hospitalization. The mean day of BSI was 38 ± 47.9 (range 3 – 323 days). Appropriate empiric antimicrobial therapy were begun within 24 hours in 52.4%. Eighteen patients (28.6%) had a Charlson index ≥ 3. The crude (overall, in hospital) mortality was 47.6%.

### Microbiological features

Of the 63 *P. aeruginosa *isolates included in this study, 73.0% of BSI were caused by imipenem-susceptible *P. aeruginosa *and 27.0% by imipenem-resistant *P. aeruginosa*.

### Mortality and severity of illness scoring systems

Median APACHE II, SAPS II and SOFA scores on the day of BSI were 23, 46 and 7, respectively. Computation of the area under the ROC curve (table 2) for APACHE II, SAPS II and SOFA was performed at days -2, -1, 0, 1 and 2 of BSI. The values for area under the ROC curve showed that the three scores had good discriminative power in the prediction of poor outcome. To evaluate the extent to which the applied scoring systems were valid for predicting mortality in patients with *P. aeruginosa *nBSI, the sensitivity, specificity, overall correctness of prediction and positive and negative predictive values were determined on the day of greatest area under the curve (day -1 of BSI for APACHE II and SAPS II, and at day +1 for SOFA). Table 3 shows the utility parameters calculated at the cut-off point that gave the best Youden index. The best Youden index and the highest positive predictive value were found for the APACHE II score. SAPS II score had the best sensitivity 93.3%; however the best specificity value was found for APACHE II score (87.9%). The best negative predictive value (90.5%) was seen with the SAPS II score. As seen in table 4, the multivariate analysis controlling for other potential predictors of outcome was done for all the scoring systems on the best discriminating day to identify the best predictor for mortality. The SAPS II threshold score was most predictive (OR: 13.03, CI95% 2.51–70.49), followed by APACHE II (OR: 12.51, CI95% 3.12–50.09), and SOFA (OR: 5.49, CI95% 1.56–19.30).

**Table 2:** Comparison between receiver operating characteristic (ROC) curves for APACHE II, SAPS II and SOFA scores at days -2, -1, 0, 1 and 2 of *P. aeruginosa *BSI.

	Area under the ROC curve				
	
**DAYS of BSI**	**-2**	**-1**	**0**	**1**	**2**
**APACHE II**	0.741	0.821	0.793	0.804	0.787
**SAPS II**	0.777	0.809	0.793	0.785	0.783
**SOFA**	0.718	0.762	0.743	0.806	0.783

**Table 3:** Prediction of subsequent hospital mortality determined on day with the best discriminating power for *P. aeruginosa *BSI.

**Scoring system**	**Day with best discriminating power**	Cut-off point*	**Youden Index**	**Sensitivity (%)**	**Specificity (%)**	**Overall correctness**	**PPV (%)**	**NPV (%)**
**APACHE II**	-1	22	0.579	70.0	87.9	26.0	84.0	76.3
**SAPS II**	-1	33	0.509	93.3	57.6	34.0	66.7	90.5
**SOFA**	+1	5	0.391	81.5	57.6	46.3	61.1	79.2

PPV = positive predictive value, NPV = negative predictive value, * = value giving the best Youden index.

**Table 4:** Stepwise logistic regression analysis for each scoring systems with crude mortality at day with the best discriminating power for *P. aeruginosa *BSI.

	**Died (n = 30)**		**Recovered (n = 33)**		**Univariate analysis**		**Multivariate analysis**	
	
**VARIABLES AT DAY -1**	N	%	N	%	OR	CI 95%	OR	CI 95%
**APACHE II >22 at day -1**	21	84.0	4	16.0	16.92	4.59–62.37	12.51	3.12–50.09
**Inadequate antibiotic therapy**	16	53.3	14	46.7	1.26	0.75–2.11		
**Imipenem resistant *P. aeruginosa***	10	58.8	7	41.2	1.86	0.60–5.74		
**Charlson score ≥ 3**	14	77.8	4	22.2	6.34	1.78–22.54	2.28	0.49–10.63

**SAPS II >33 at day -1**	28	66.7	14	33.3	19.00	3.87–93.36	13.03	2.51–70.49
**Inadequate antibiotic therapy**	16	53.3	14	46.7	1.26	0.75–2.11		
**Imipenem resistant *P. aeruginosa***	10	58.8	7	41.2	1.86	0.60–5.74		
**Charlson score ≥ 3**	14	77.8	4	22.2	6.34	1.78–22.54	2.50	0.63–9.90

	**Died (n = 27)**		**Recovered (n = 33)**		**Univariate analysis**		**Multivariate analysis**	
	
**VARIABLES AT DAY +1**	N	%	N	%	OR	CI 95%	OR	CI 95%

**SOFA > 5 at day + 1**	22	61.1	14	38.9	5.97	1.81–19.66	5.49	1.56–19.30
**Inadequate antibiotic therapy**	14	50.0	14	50.0	0.68	0.25–1.90		
**Imipenem resistant *P. aeruginosa***	9	56.3	7	43.8	1.86	0.58–5.90		
**Charlson score ≥ 3**	12	75.0	4	25.0	5.80	1.59–21.11	5.23	1.31–20.90

## Discussion

*Pseudomonas aeruginosa *remains an important cause of BSI and one of the principal pathogens responsible for severe organ dysfunction. The present study compares the three most useful severity of illness scoring systems applied in studies of bloodstream infection. We decided to compare APACHE II, SAPS II and SOFA on the day of bacteremia and two days prior to positive blood culture through two days afterwards in order to determine which scoring system was most predictive for mortality.

The ability to assess a complex clinical condition such as bloodstream infection, using relatively simple scores may facilitate communication with regards to the severity of the physiologic process. Our intention is to improve the assessment of *P. aeruginosa *BSI so as to identify different patterns of organ dysfunction, and thereby enhance our understanding of the infectious process, as well as gaining knowledge of a patient's prognosis. BSIs should not be seen as static phenomena, but rather as a continuum of alterations with changes in the patients' condition seen daily. Many intrinsic variables are involved (e.g., underlying diseases, age, and gender), but interventional variables such as adequate initial antibiotic treatment [13], rapid removal of catheters responsible for perpetuating the infection [14], and optimization of hemodynamic status with fluid resuscitation or vasopressors [15,16] may determine the evolution of this process. If we had calculated the prognostic scores on our patients at the time of hospital admission, given that one half of the patients acquired BSI after 21 days of hospitalization, the prediction of mortality would likely be totally different and would not take into account the impact of the nosocomial bloodstream infection.

Many intensive care units receive trauma and surgical patients and they are able to stratify acutely ill patients prognostically with success by using APACHE II [4], SAPS II [6] or SOFA scores [7]. However the proportion of infected patients in these studies of scoring systems was less than 30%. To our knowledge, until this point there has been no study that compares the severity of illness scoring systems for patients with nosocomial bloodstream infection. In patients with *P. aeruginosa *BSI, calculation of the area under the ROC curve in our study confirmed that assessing scores at day -1 was best for SAPS II and APACHE II, while SOFA was best assessed on day +1 of BSI. Multivariate analysis was performed to control for underlying disease using the Charlson weighted comorbidity index. This confirmed that SAPS II and APACHE II on day -1 were the best predictors for mortality (Table 4).

In the APACHE II and SAPS II studies, calculations were done only on the day of admission [4,6]. A previous report showed that the APACHE II score at admission was not useful as a prognostic factor, whereas the progression of organ dysfunction after the onset of pneumonia due to *P. aeruginosa *in intubated patients was predictive [5]. However, other studies showed increased APACHE II scores as a risk factor for mortality [3,10]. On the other hand, in the SOFA study the scores were calculated every day [7]. Other studies comparing scoring systems in cirrhotic patients with renal failure found that the discriminatory power of SOFA for predicting mortality was superior to the other scoring systems [17].

The limitations of our study should be acknowledged. First, we performed a retrospective study. Second, we applied the APACHE II and SAPS II scores daily although these scores were originally intended to be calculated after the first 24 hours of ICU care. And lastly, it is a single center study which limits its generalizability.

## Conclusion

In conclusion, the crude mortality rate is high for *P. aeruginosa *BSI. The data in this study demonstrate that SAPS II and APACHE II are more accurate than SOFA scores for predicting mortality in this group of patients. Retrospectively calculating the APACHE II or SAPS II score on the day preceding the first blood culture yielding *P. aeruginosa*, could be helpful to clinicians in discussing prognosis with patients' families. Furthermore, these data can guide future studies that further delineate the epidemiology of nosocomial BSI, including efforts to determine which variables may be associated with improved outcome among patients with nosocomial BSI.

## Competing interests

The author(s) declare that they have no competing interests.

## Authors' contributions

ARM participated in the design of the study, collected the data and performed the statistical analysis. GMLB participated in the design of the study and performed the statistical analysis. RPW participated in the design of the study and coordination. MBE conceived of the study, and participated in its design and coordination. All authors read and approved the final manuscript.

## Pre-publication history

The pre-publication history for this paper can be accessed here:


